# Modelling optimal allocation of resources in the context of an incurable disease

**DOI:** 10.1371/journal.pone.0172401

**Published:** 2017-03-13

**Authors:** Betty Nannyonga, David J. T. Sumpter

**Affiliations:** 1 Department of Mathematics, School of Physical Sciences College of Natural Sciences, Makerere University, Kampala, Uganda; 2 Department of Mathematics, Center for Interdisciplinary Mathematics Mathematics Institution, Uppsala University, Uppsala, Sweden; University College London, UNITED KINGDOM

## Abstract

Nodding syndrome has affected and led to the deaths of children between the ages of 5 and 15 in Northern Uganda since 2009. There is no reliable explanation of the disease, and currently the only treatment is through a nutritional programme of vitamins, combined with medication to prevent symptoms. In the absence of a proper medical treatment, we develop a dynamic compartmental model to plan the management of the syndrome and to curb its effects. We use incidence data from 2012 and 2013 from Pader, Lamwo and Kitgum regions of Uganda to parameterize the model. The model is then used to look at how to best plan the nutritional programme in terms of first getting children on to the programme through outreach, and then making sure they remain on the programme, through follow-up. For the current outbreak of nodding disease, we estimate that about half of available resources should be put into outreach. We show how to optimize the balance between outreach and follow-up in this particular example, and provide a general methodology for allocating resources in similar situations. Given the uncertainty of parameter estimates in such situations, we perform a robustness analysis to identify the best investment strategy. Our analysis offers a way of using available data to determine the best investment strategy of controlling nodding syndrome.

## Introduction

In the northern part of Uganda, thousands of children have fallen ill with a little known, incurable nodding disease syndrome, NDS. Communities are panicking and losing hope as neither the cause nor a cure are known [1]. The affected Ugandan districts include Kitgum, Lamwo, Pader and Gulu. The nodding syndrome emerged in Sudan in the 1960s [2], although it was first reported in 1962 in secluded mountainous regions of Tanzania [3]. Since then, it has been reported in small regions in South Sudan, Tanzania and Uganda [4, 5]. However, the connection between that disease and the nodding syndrome was just made recently [5].

Nodding disease affects young children between the ages of five to fifteen. It is mentally and physically disabling, eventually fatal. As of 2013, there were more than 3,094 cases and 170 deaths in northern Uganda [6]. Between 2012 -2013, the government of Uganda set up outreach and screening centers at health facilities but due to deep poverty, many families could not afford to pay for transport to these facilities. Children affected by NDS are characterized by complete and permanent stunted physical growth [7]. The brain is also stunted, leading to mental retardation of the symptomatic individual. Due to pathological bobbing of the head, the disease is named nodding syndrome. The nodding was discovered to be an atonic seizure [8], which often begins when children eat or feel cold. The seizures are brief and stop when the child stops eating or feels warm again and they can manifest with a wide degree of severity. When severe, the affected child can collapse, which may result in further injury [9], such as falling into the open fire. Although inconclusive, there is documented association of the disease with malnutrition and *onchocerciasis*. NDS is a progressive fatal disorder with a duration of about three years or more [10]. A small fraction of the symptomatic survive, and the majority dies [7].

Nodding disease started spreading across the Sudan-Uganda border in 2009 where more than 2000 cases [3] were reported in Uganda’s Kitgum district [4]. By the end of 2011, NDS outbreaks were concentrated in Kitgum, Pader and Gulu [11]. The spread and manifestation of outbreaks may be made worse by the poor health care of the region [10]. Children suffering from nodding disease syndrome are exposed to poverty and malnutrition, creating a fresh distress to the already suffering region [12], and exposing children to otherwise preventable deaths. The recent results from CDC [13] report crystals found in victims’ brains. Still, there are no answers to why the illness attacks only children, and in most cases, some and not all in a family. Further, there is no answer of whether there is a genetic predisposition. The impact of NDS has been highly destructive and health officials are left mystified as to cause and cure. Children living under the poorest conditions with insufficient food, no safe drinking water, or indecent housing are most susceptible to this condition. In addition, the affected children are often neglected by their parents [14] and barred from schools [15].

The outreach and screening centers set up by the Uganda government provide nourishing foods and medication for symptoms to the patients. Each month, patients are examined to determine any improvement in the level of malnutrition, frequency and severity of seizures. The nutritional programme is aimed to minimize the symptoms such as occurrences of seizures. However, resources are extremely limited. The research question we address is how to balance the use of resources in getting children on to a nutritional programme and symptom preventing medicines in the first instance through *outreach*, against keeping them on this treatment programme once they have started, through *follow-up*. In many Uganda health contexts, outreach means that services that are not easily available are taken nearer to the populace. Once these services are set up, getting the children to go to the centers involves community effort through local councils visiting their respective villages and urge the next of kin to take them there. Follow-up is when children return the following month for check up and additional food supplements where required. In the absence of any cure in the foreseeable future, we look at how to best utilize the limited available resources to minimize the impact of the disease. We address this problem using a mathematical model.

Mathematical models have been used for optimization of disease prevention [16–19] and epidemic control [20–26]. The models enable more efficient searches for desirable control strategies by considering all strategies simultaneously. The results direct the models in generating detailed predictions of potential future outbreaks. An optimization model on foot-and-mouth disease [27], was used to evaluate alternative control strategies that minimized the total regional cost for the entire epidemic duration, given disease dynamics and resource constraints. A similar model using optimal control theory was developed for dengue fever [28] to analyze the optimal strategies for using these controls, and respective impact on the reduction/eradication of the disease during an outbreak in the population. A model was designed to investigate problems of disease prevention and epidemic control, in which two sets of decisions namely vaccinating individuals and closing locations were optimized with the goal of minimizing the expected number of symptomatic individuals after intervention [29]. Similar to our objective, the goal of the paper was to formulate mathematical optimization models, to identify which subset of individuals should be vaccinated and which locations to close in order to minimize the expected number of symptomatic individuals. Results of such models have proved beneficial in decision and policy making on management of disease outbreaks and prevention.

In our model, three states are used: the symptomatic, treated and stunted. The symptomatic children that have not been exposed to any form of treatment or food supplements. The treated, are those that have received medication for symptoms of nodding syndrome including malnutrition, seizures, and poor growth. The stunted are the children whose growth has been inhibited due to nodding disease. These have either never received any medication or food supplements, or have dropped out of treatment. Using the model, we determine the optimal percentage of investment in treatment vs follow-up, with the overall objective of minimizing stunted growth of the affected children.

### Available data

The data used in this study was collected from Pader and Lamwo districts in the northern part of Uganda between 2012 and 2013 by the Ministry of Health Uganda. Outreach centers were established in the districts and serviced villages in respective parishes and sub-counties. A team consisting of twenty (20) personnel from the Ministry of Health visited children once every month in ten (10) different centers where each health official worked for six (6) hours from 10:00 am to 4:00 pm, giving a total of 1200 man hours. In order to operationalize this data to use it in the model, for each the 994 patients, we recorded

1. Age at first visit;
2. Date of initial onset;
3. How many times the patient accessed medical treatment again within six months;
4. The home villages of each of the patients.

Children were brought into the health centers by their parents or next of kin who were informed of the study and verbal consent obtained to treat, evaluate and follow-up. On their first evaluation, history and physical assessment of the affected children was done and recorded. The healthcare officials used the gathered information to determine the severity of the syndrome and level of malnutrition for each patient. In severe cases of acute malnutrition, food supplements including ready-to-use- therapeutic foods (RUTF) were given to control undernutrition and prevent death. Sodium valproate (INN), an anticonvulsant used in the treatment of epilepsy, was given to control the atonic seizures. On subsequent visits, individuals were assessed on whether they were taking the drugs consistently or if they defaulted. The data used in this study was authorized by the Ministry of Health Uganda who approved this retrospective study. The records used in the study were anonymized and de-identified prior to analysis.

### Model formulation and analysis

**Fig 1:**
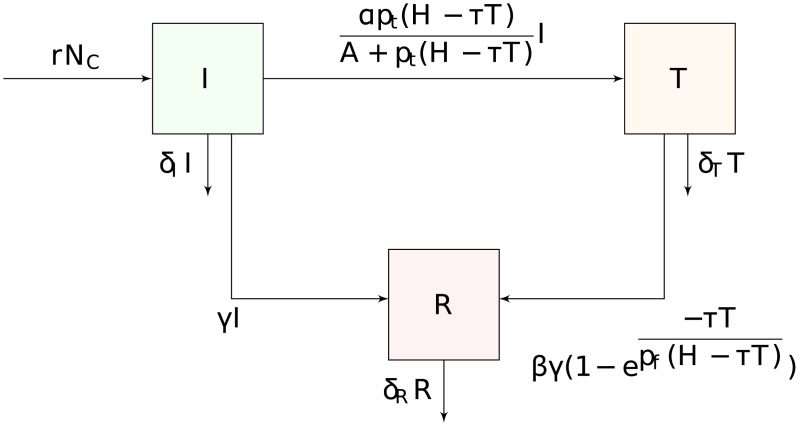
Flow diagram for the dynamics of nodding syndrome.

We consider a population of children with and without nodding syndrome. Children with the syndrome are divided into three sub groups depending on whether they seek medication and follow-up or not. Subgroup *I* gives the total number of children, who were previously healthy, but currently with malnutrition, reported head nodding and seizure episodes. Subgroup *T* are the children enrolled by the healthcare officials to receive monthly medication for displayed symptoms and/or food supplements in case of malnutrition. Children in *R* have stunted growth, classified based on visible growth impairment that was determined using the current height as compared to previous determinations and body weight corresponding to current age. The children are impaired due to absence of or fall-out from treatment and malnutrition. More details are shown in Fig 1.

Nodding syndrome has been reported to exist in children aged between 5–15 years [2, 30, 31]. From the data, the minimum age at which a child was recorded to have nodding syndrome symptoms was 2 and the maximum 27. However, out of the 994 individuals recorded, 947 (95%) are between the ages of 5–16. Therefore, the age group of 5–16 is used to estimate the rate at which children develop nodding syndrome. A child enters subgroup *I* at a rate *r*, when they start to display nodding syndrome symptoms such as head bobbing, seizures and malnutrition. Prior studies have given the prevalence of the nodding syndrome in Kitgum district to be 12 per 1000 [30, 31]. We shall use the same value for Pader and Lamwo since they are in the same geographical region sharing borders. In order to transform this estimate into a rate per month we divide by the age range over which the disease can occur and the months in a year, i.e. *r* = 12/(1000 × 11 × 12) = 0.00009. The rate of development of nodding syndrome is a function of the total number of children *N*_*C*_, in each district. In Kitgum, 45,800 children were reported in the age range of 5–15. The total population of Kitgum is 247,800, implying that 45,800/247,800 ≈ 18.48% is the percentage of susceptibles. We estimate *N*_*C*_ for Lamwo and Pader by taking 18.48% of their total populations. The population of Lamwo is 171,300, and 231,700 for Pader [32], giving the estimated number of children in these two districts as *N*_*C*_ = 0.1848 × (171,300 + 231,700) ≈ 74,474. Therefore, the number of incident cases that develop nodding syndrome per month *rN*_*C*_ = 6.7027.

The interest of this study is to minimize the number of children that develop stunted growth. We can do this by moving children from compartment *I* to *T*, and prevent movement to *R*. Children from subgroup *I* can move to subgroup *T* if they seek treatment, or *R* if they do not seek medication for the symptoms. Movement from *I* to *R* occurs when children develop stunted growth due to lack of medication for displayed symptoms. We assume that on average, a child becomes removed due to NDS after six months from onset. Therefore, the untreated development rate of nodding syndrome *γ* = 1/6. According to the data, of the 994 recorded cases, 703 accessed medication and nutritional supplements at least twice in six months. Therefore, the proportion that did not access medication at least twice in six months is 291/994 = 0.2928. This gives the rate of fall-out from medication per month as *β* = 0.2928/2 = 0.1464.

The movement from *I* into *T* depends on whether children seek medication, which in turn depends on the outreach effort by healthcare personnel. Movement from *T* to *R* depends on the efficiency of follow-up by medical personnel. In the absence of follow-up activities, children can start to display nodding symptoms. Resources for outreach and follow-up are limited, and medication for existing cases is prioritized. Professionals from the Ministry of Health set up ten (10) health centers in each district, where they visited each center and attended to the children once every month. The total number of hours they invested per month can be estimated from the data using the number of hours each healthcare official used on each visit at each center. Since 20 healthcare providers visited the districts for 6 hours a day (from 10:00am to 4:00pm), 10 times per month, the total available man hours per month is *H* = 20 × 6 × 10 = 1200.

Of the total available man hours per month *H*, a proportion is used for treatment of existing cases. We estimate the time taken to handle one child identified with nodding syndrome and in treatment to 30 minutes [33, 34]. Thus each treatment takes *τ* = 30/60 = 0.5 hours. There is one visit per treated case per month and therefore the total hours per month used for treatment is *τT*. As a result, the total available hours for outreach or follow-up is *H* − *τT*.

Of the total remaining hours after treatment *H* − *τT*, a proportion can be invested in outreach and some in follow-up. Let *p*_*t*_ be the proportion of hours dedicated to outreach activities for getting children in treatment, and *p*_*f*_ for the proportion of hours dedicated to follow-up, with *p*_*t*_ + *p*_*f*_ = 1. Then, the total number of hours used for outreach that gets children into treatment is *p*_*t*_(*H* − *τT*) per month, with *p*_*f*_(*H* − *τT*) hours used for follow-up. We do not set *p*_*t*_ at this point, but rather aim to find a value of *p*_*t*_ that gives the best strategy to invest in getting and keeping children into treatment.

In order to optimize *p*_*t*_ and *p*_*f*_, we have to make modelling assumptions about how effort is translated into success. For the transition rate from *I* to *T* the rate can be anything from 0 if no hours are invested in outreach to 1, for a situation where healthcare is widely available, and symptomatic children enter treatment within one month of displaying symptoms. We set a constant *α* = 1 to give the saturation level of outreach when at its most effective. The transition function of children from state *I* to *T* can then be written as

$$\alpha \frac{p_{t}\left(H-\tau T\right)}{A+p_{t}\left(H-\tau T\right)}$$

so that it increases with investment in outreach. *τ* is the time taken to handle one child per visit, set to 30 minutes [33, 34] and *A* is the half saturation constant, which is the point where children from at least 50% of the villages received medication. This value can be estimated by taking 50% of the total number of villages in the data (211), giving *A* = 50% × 211 = 105.5.

For the transition rate from *T* to *R*, a child would fall-out from treatment and develop stunted growth at a rate *β*. The fall-out rate can be close to 1 when more hours are invested in getting children into treatment and close to 0 if follow-up care of at least twice in six months is available. The constant *β* = 0 signifies the success and effectiveness of follow-up care. The transition function of children from state *T* to *R* is given as

$$\beta \gamma \left(1-\text{exp}(-\tau T/\left(p_{f}\left(H-\tau T\right)\right))\right),$$

and is large if investment in follow-up is small.

With the descriptions of *I*, *T* and *R*, we can assume that *δ*_*R*_ > *δ*_*I*_ > *δ*_*T*_ since children in *T* are receiving medication and follow-up and therefore die at a slower rate than *I*, whereas children in *R* are stunted and sometimes brain dead due to the numerous seizures and more often die faster from other accidents such as falling in open fires during abrupt seizures. With outreach, medication and follow-up, symptomatic children recover within a period of three years (*d* = 3 × 12 = 36 months) or more, or die from the symptoms [10]. With the assumption that 36 months is the time an individual should recover or die from the nodding syndrome, we set the disease induced death rates for *I* to *δ*_*I*_ = 1/36, for *T* to *δ*_*T*_ = 0.5 × *δ*_*I*_, and *R* to *δ*_*R*_ = 1.5 × *δ*_*I*_. These modification parameters are guesses that respectively inhibit or enhance death rates with or without medication. We summarize all the parameters discussed in Table 1, and give a set of equations describing the process in Eq (1).

**Table 1:** Parameter estimates from Uganda data.

Parameter	Description	Dimensions
*r*	Development rate	0.00009 / month
*N*_*C*_	Number of children	74,474
*γ*	Stunted growth development rate	1/6/month
*β*	Medication fall-out rate	0.1464 /month
*H*	Total man hours per month	1200 man hours/month
*τ*	Number of hours per visit per child	0.5 hours/visit/month
*p*_*t*_	Proportion in treatment	[0,1]
*p*_*f*_	Proportion in information	[0, 1 − *p*_*t*_]
*α*	Rate at which treatment starts in ideal situation	1/month
*A*	Half saturation constant	105.5
*d*	Duration of nodding syndrome before recovery/death	36 months
*δ*_*I*_	Disease induced death rate of symptomatic	1/36 /month
*δ*_*T*_	Disease induced death rate of those on treatment	0.5/36 /month
*δ*_*R*_	Disease induced death rate of the stunted	1.5/36 /month

Parameter estimates for Lamwo and Pader.

From the above descriptions and assumptions, we can now write the system of equations describing the system as follows:

$$\begin{array}{ccc} \frac{dI}{dt} & = & rN_{C}-\frac{\alpha p_{t}\left(H-\tau T\right)}{A+p_{t}\left(H-\tau T\right)}I-\gamma I-\delta_{I}I,\\ \frac{dT}{dt} & = & \frac{\alpha p_{t}\left(H-\tau T\right)}{A+p_{t}\left(H-\tau T\right)}I-\beta \gamma \;\left(1-e^{\frac{-\tau T}{p_{f}\left(H-\tau T\right)}}\right)-\delta_{T}T,\\ \frac{dR}{dt} & = & \gamma I+\beta \gamma \;\left(1-e^{\frac{-\tau T}{p_{f}\left(H-\tau T\right)}}\right)-\delta_{R}R. \end{array}$$ (1)

To analyze the model in Eq (1), we start by transforming it using the following approximations: If the proportion of investment in getting children in treatment is very small, (i.e. small *p*_*t*_(*H* − *τT*)), *αp*_*t*_(*H* − *τT*)*I*/(*A* + *p*_*t*_(*H* − *τT*)) can be approximated to *αp*_*t*_(*H* − *τT*)*I*/(*A*). Small investment in getting children in treatment implies big investment in follow-up. Therefore, when *p*_*t*_(*H* − *τT*) is very small, *p*_*f*_(*H* − *τT*) is very big. At this point, *βγ*(1 − exp(*τT*/(*p*_*f*_(*H* − *τT*)))) tends to *βγτT*/(*p*_*f*_(*H* − *τT*)). Since *p*_*t*_ + *p*_*f*_ = 1, we make the substitution *p*_*f*_ = 1 − *p*_*t*_ and approximate Model (1) to

$$\begin{array}{ccc} \frac{dI}{dt} & = & rN_{C}-\frac{\alpha p_{t}\left(H-\tau T\right)}{A}I-\gamma I-\delta_{I}I,\\ \frac{dT}{dt} & = & \frac{\alpha p_{t}\left(H-\tau T\right)}{A}I-\frac{\beta \gamma \tau }{\left(1-p_{t}\right)\left(H-\tau T\right)}T-\delta_{T}T,\\ \frac{dR}{dt} & = & \gamma I+\frac{\beta \gamma \tau }{\left(1-p_{t}\right)\left(H-\tau T\right)}T-\delta_{R}R. \end{array}$$ (2)

In terms of the treated T*, the equilibrium points are given by

$$\begin{array}{cc} I^{*} & =\frac{A}{\alpha p_{t}\left(H-\tau T^{*}\right)}\left(\frac{\beta \gamma \tau }{\left(1-p_{t}\right)\left(H-\tau T^{*}\right)}+\delta_{T}\right)\;T^{*},\\ R^{*} & =\frac{1}{\delta_{R}}\left[\left(\frac{\gamma A}{\alpha p_{t}\left(H-\tau T^{*}\right)}\right)\left(\frac{\beta \gamma \tau }{\left(1-p_{t}\right)\left(H-\tau T^{*}\right)}+\delta_{T}\right)+\frac{\beta \gamma \tau }{\left(1-p_{t}\right)\left(H-\tau T^{*}\right)}\right]\;T^{*},\\ 0 & =rN_{C}-\frac{AT^{*}}{\alpha p_{t}\left(H-\tau T^{*}\right)}\left(\frac{\alpha p_{t}\left(H-\tau T^{*}\right)}{A}+\left(\gamma +\delta_{I}\right)\right)\left(\frac{\beta \gamma \tau }{\left(1-p_{t}\right)\left(H-\tau T^{*}\right)}+\delta_{T}\right). \end{array}$$ (3)

Due to complexity of the model, analysis can be done numerically to obtain mathematical conclusions by simulating the steady states for different values of *p*_*t*_ ∈ [0, 1]. We assume that once the children with nodding syndrome are identified, they all opt for treatment within a month. This assumption helps us to obtain the point for optimal investment in the benefit of treatment and information. The optimal value of *p*_*t*_ corresponds to the best strategy of investment and gives the minimum number of patients that develop stunted growth or equivalently the maximum number enrolled for medication.

**Fig 2:**
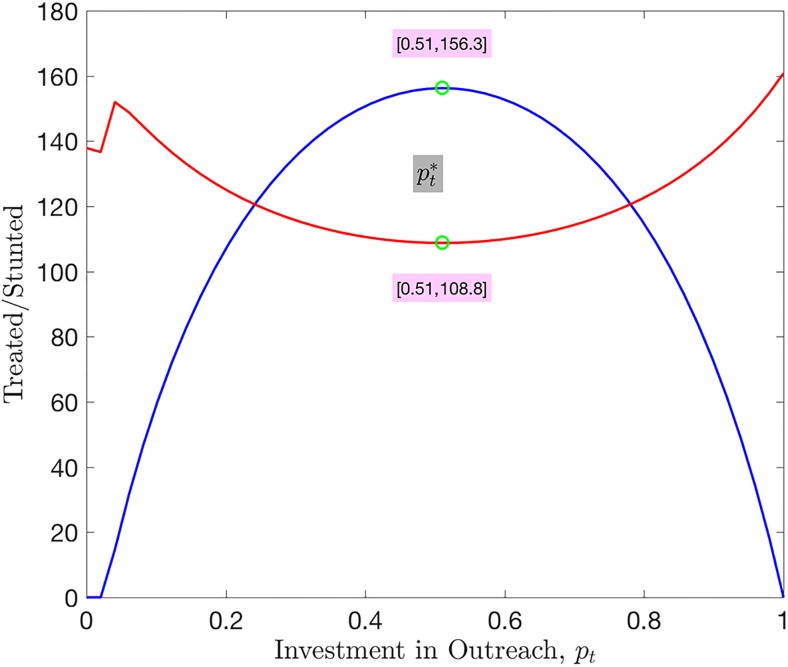
Simulation of the steady states with *p*_*t*_ ∈ [0, 1]. The red line is for the stunted while the blue line is for the children on treatment. Note that maximum *T* (minimum *R*) is achieved at the point *p*_*t*_ = 0.51. This gives $p_{t}^{opt}.$ The parameter values used are given in Table 1.

Fig 2 (blue line) shows that at the beginning of investment in outreach (about 2%), there is no significant increase in the number of children enrolled in treatment. However, the red line representing the stunted children shows a slight fall in the number of stunted growth followed by a sudden jump, and then later to a steady decrease to a minimun value attained when optimal investment is achieved. With further outreach, the blue curve shows the point at which the number of children on treatment *T* increase to a maximum. This corresponds to the optimal percentage of investment in outreach, (that is, getting children into treatment) $p_{t}^{opt}=0.51$ above which further investment does not yield payoff as the number of children in treatment begin to go down. This is due to the fact that children with nodding syndrome need regular follow-up visits for food supplements and an anticonvulsant to control the atonic seizures. Without follow-up, there is no evident improvement, and this leads could lead to less children enrolling for medication and supplements. The red curve (representing stunted children) shows the point when *R* is minimum obtained when $p_{t}=p_{t}^{opt}.$ This is the point when $p_{f}^{min}=1-p_{t}^{opt}$ below which investment in follow-up does not prevent children from dropping out of treatment. It is observed from the red curve that increase in *p*_*t*_ corresponds to a reduced *p*_*f*_ and results in increase in stunted growth *R*. These results are obtained under the assumption that healthcare is widely available, and symptomatic children enter treatment within one month of displaying symptoms, to give the saturation level of outreach when at its most effective.

**Fig 3:**
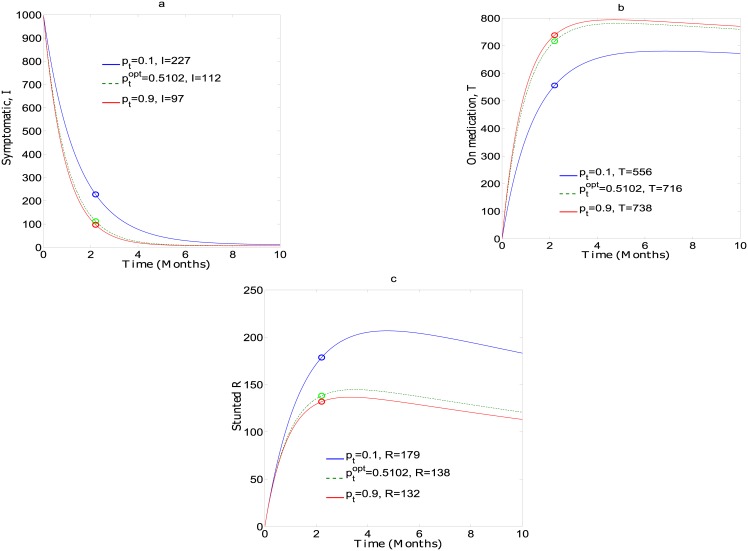
Simulation of the model for different values of *p*_*t*_ ∈ [0, 1], and initial conditions *I* = 1,000, *T* = 0, and *R* = 0. Parameter values used are in Table 1.

Using the value of $p_{t}^{opt}$ obtained from Fig 2, the model is simulated to study the behavior of states *I*, *T* and *R* around this point for $p_{t}<p_{t}^{opt}$ and $p_{t}>p_{t}^{opt}.$ Fig 3 shows the change in the number of symptomatic children *I*, those enrolled in treatment *T* and the stunted or removed *R* when *p*_*t*_ = 0.1, 0.51, and 0.9. It is observed from the figure that the point $p_{t}^{opt}$ is obtained after 2 months. This implies that it gets harder to keep children in treatment after two successive visits, and further investment without keeping those enrolled does not prevent stunted growth. The number of children after two months of treatment with the different levels of investment in outreach is approximately *I*(0.10) = 227, *I*(0.51) = 112, and *I*(0.90) = 97. We see that the jump in the number of children on treatment from 10% to 51% investment is 115, while that from 51% to 90% is 15. Similarly, *T*(0.10) = 556, *T*(0.51) = 716, and *T*(0.90) = 738; and *R*(0.10) = 177, *R*(0.51) = 138, and *R*(0.90) = 132, with big jumps from *T*(0.10) to *T*(0.51) as compared to *T*(0.51) to *T*(0.90). Thus when optimal investment in treatment is attained further input does not yield significant benefits.

To obtain $p_{t}^{opt}$, it was assumed that the treatment rate *α* is 1, while the rate of stunted development after fall-out from treatment *β* is 0.1464. However, we can obtain different optimal investment strategies for different sets of combinations of *α* and *β* to minimize stunted growth. This is done by simulating the model for *α*, *β* ∈ [0, 1] to determine when to invest in getting children into treatment and when to switch to follow-up.

**Fig 4:**
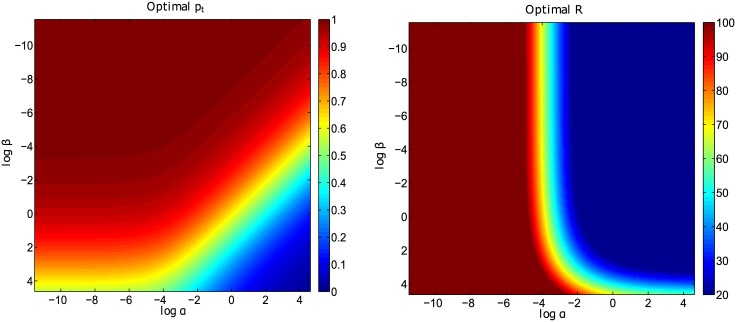
Simulation of the model for different values of *α*, *β* ∈ [0, 1]. (a) Values of *p*_*t*_ for all *α* and *β* combinations; (b) Corresponding values of *R*. Parameter values used are in Table 1.

Fig 4a and 4b shows the results of log(*α*) simulated against log(*β*) for optimal investment in getting children into treatment *p*_*t*_ and the respective number of children who develop stunted growth *R*. It is observed from the figure that when the treatment rate *α* is large, it does not payoff to invest more in getting children in treatment and we should optimize follow-up. The value of *p*_*t*_ that minimizes stunted growth depends on *α* and *β*. For most of the *α* and *β* values, *p*_*t*_ is large indicating that investment in getting children into treatment does not prevent stunted growth. These results are observed over a wide range of *α* and *β* although direct investment in getting children into treatment is optimal when *α* is small and *β* large.

## Discussion and conclusion

Nodding syndrome has lasted a period of over five years in Pader and Lamwo districts in northern Uganda killing mainly children between 5 and 15 years [2, 30, 31]. As a result, more than 3,094 children are symptomatic and over 170 dead, in the war torn region with extreme poverty. This paper provides a possible strategy of how limited resources can be utilized to optimize control of the disease, first by getting children into treatment, and secondly, keeping them there. We hypothesize that getting children into treatment is necessary but keeping them there is sufficient to prevent the affected children from stunted growth. We design a mathematical model parameterized using data from Pader and Lamwo districts of Uganda, to provide a reliable optimal investment strategy that prevents stunted growth. The model is used to show that if all children access treatment within a month of displaying symptoms, then up to 51% of available resources should be invested in getting children into treatment and not less than 49% should be put in follow-up. Fig 2a shows that there is a maximum number of children in treatment at the optimal point, suggesting that further investment would not be economical. Fig 2b shows that as investment into medication is increased, the number of stunted children reduce. At the point when optimal investment into getting children into treatment is attained, we obtain the minimum number of stunted children; beyond this point, the number of children that develop stunted growth begin to increase. This is the point when to switch from further investment in treatment to investing into follow-up. Looking at the number of symptomatic children after two months with no investment (*p*_*t*_, *p*_*f*_ = 0,) gives 689. Without medication, these will eventually develop stunted growth and die. If all investment is put in follow-up (that is, *p*_*f*_ = 1, *p*_*t*_ = 0), there are 266 stunted growth which is a 61% reduction. However, if 51% is invested in outreach (*p*_*t*_ = 0.51, *p*_*f*_ = 0.49), there is a reduction of 80% stunted growth. From this we conclude that the best strategy is to invest about half in outreach, and half on follow-up and predicts that the percentage number of stunted growth cases will be 80% less following this strategy compared to the simple strategy of all follow-up.

With the value of optimal *p*_*t*_ obtained in Fig 2a, we simulated the model in Eq (1) to determine change if any, when values of $p_{t}<p_{t}^{opt}$ and $p_{t}>p_{t}^{opt}$ are used. Fig 3a, 3b and 3c showed the change in symptomatic, treated and stunted children with these values. Fig 3 indicates that any investment strategy with a value of $p_{t}>p_{t}^{opt}$ is not economical and does not yield any benefits. This is the point at which a switch from outreach to follow-up should be made.

ure To further determine the best investment strategy, we simulated the steady states while varying the rate *α*, at which treatment starts in an ideal situation, and the treatment fall-out rate *β*. Fig 4 postulates that when the symptomatic children access treatment immediately after onset of symptoms and the fall-out rates are low, more investment should be put in getting more children into treatment (Fig 4). If the children enroll in treatment immediately but the fall-out rates are very high, then there should be little or no investment (0–20%) in getting children into treatment; most investment (80–100%) should be utilized for follow-up. However, if the children take very long to access treatment and the fall-out rates are very high, then up to 60% should be utilized to get more children into treatment and about 40% into follow-up. When access to treatment is slow with low fall-outs, then all resources should be invested into getting more children into treatment. It is important to note that the fall-out rates determined the investment strategy for our model. Fast access to treatment coupled with high fall-out rates call for more investment into follow-up (0–20%), while slow access to treatment with high fall-out rates call for about 55% investment into treatment and 45% into follow-up. Fig 4 shows that when both *α* and *β* are high, that is, fast access to treatment and high fall-out rates, it is not optimal to further invest into getting children into treatment, since most of them are in treatment. Therefore, it is more economical to invest in follow-up to reduce the high fall-out rates and minimize the development of stunted growth.

This study provides the best way of utilizing limited resources to effectively control nodding syndrome and minimize deaths and further development and stunted growth. The results we obtained here show that prevention of stunted growth depends on two parameters; how fast a child accesses medication *α*, and the probability of remaining on treatment *β*. In most cases, when access to medication and fall-out rates are low, we should make greater investment in getting more children into treatment than in follow-up, i.e optimal *p*_*t*_ remained high for all low values *α*, and more children developed stunted growth for all values of *β* and *α* < 11.85%. In most cases, optimal investment into getting children into treatment *p*_*t*_ is 100%, except for the instances when fall-out rate *β* is very high (>4), and access to treatment rate *α* is very small (<7.29%). The results show that there is a limit to how much you can invest in getting children in treatment and keep them there without developing stunted growth. In the nodding case discussed here, local council representatives visited homes with suspected children and urged their next of kins to take them to screening and outreach centers for medication and management information. The key study limitation was not knowing the onset of symptoms of nodding syndrome such that initial dates were taken to be when parents suspected their children of having nodding disease after repeated seizures and nodding. The approach we use here can help control nodding children from developing stunted growth especially when faced with limited information and show what the benefits of outreach and follow-up against nodding syndrome could have been. Moreover our conclusions are robust against the most uncertain parameters in our model such as *δ*_*T*_ and *δ*_*R*_. Doubling or halving these values preserves the advantage of the mixed strategy over the single strategies. However, other diseases that have been neglected due to limited resources could be controlled in a similar way.

## Supporting information

S1 Fig (PDF)

S2 Fig (PDF)

S3 Fig (PDF)

S4 Fig (PDF)

S1 File (PDF)

S2 File (PDF)

S3 File (PDF)

S4 File (PDF)

S5 File (PDF)
