# Supplementary material for: Modelling optimal allocation of resources in the context of an incurable disease
Source: PLoS One. 2017 Mar 13;12(3):e0172401. doi: 10.1371/journal.pone.0172401 (PMC5347997; doi:10.1371/journal.pone.0172401)
Supplement: S1 Fig — (PDF) [file pone.0172401.s001.pdf]

Flow diagram for the dynamics of nodding syndrome
