# Supplementary material for: Modelling optimal allocation of resources in the context of an incurable disease
Source: PLoS One. 2017 Mar 13;12(3):e0172401. doi: 10.1371/journal.pone.0172401 (PMC5347997; doi:10.1371/journal.pone.0172401)
Supplement: S2 Fig — (PDF) [file pone.0172401.s002.pdf]

Simulation of the steady states with $p_t \in [0,1].$ The red line is for the stunted while the blue line is for the children on treatment. Note that maximum T (minimum R) is achieved at the point $p_t=0.51.$ This gives $p_t^{opt}.$ The parameter values used are given in Table \ref{tab:2}.
