# Supplementary material for: Modelling optimal allocation of resources in the context of an incurable disease
Source: PLoS One. 2017 Mar 13;12(3):e0172401. doi: 10.1371/journal.pone.0172401 (PMC5347997; doi:10.1371/journal.pone.0172401)
Supplement: S4 Fig — (PDF) [file pone.0172401.s004.pdf]

Simulation of the model for different values of $\alpha, \beta \in [0,1]$; (a) Values of p_t for all α and β combinations; (b) Corresponding values of R. Parameter values used are in Table \ref{tab:2}.
