# Supplementary material for: Modelling optimal allocation of resources in the context of an incurable disease
Source: PLoS One. 2017 Mar 13;12(3):e0172401. doi: 10.1371/journal.pone.0172401 (PMC5347997; doi:10.1371/journal.pone.0172401)
Supplement: S1 File — (PDF) [file pone.0172401.s005.pdf]

<u>**LINE LIST FOR SUSPECTED NODDING/EPILEPSY DISEASE ADMITTED IN KITGUM DISTRICT**</u>

	Head Nodding		Fits		Stunted Growth		Wasting		Skin Change		Nodules		In School		Out of School		School	Remarks
	y	N	Y	N	Y	N	Y	N	Y	N	Y	N	Y	N	y	N		
1		N	Y			N		N		N		N		N	y			
2	y		Y		Y	N		N		N		N		N	y			
3	y		Y		Y		Y			N		N		N	y			
4	y		Y		Y		Y		Y			N		N	y			
5	y		Y		Y		Y			N		N		N	y			
6	y		Y		Y		Y		Y			N		N	y			
7	y		Y		Y		Y			N		N		N	y			
8	y		Y		Y		Y		Y			N		N	y			
9	y																	
10	y																	
11	y																	
12	y		Y		Y													

13	y		Y		Y		Y			N		N		N	y			
14	y		Y		Y		Y			N		N		N	y			
15	y		Y		Y		Y			N		N		N	y			
16	y		Y		Y		Y			N		N		N	y			
17	y		Y		Y		Y			N		N		N	y			
18	y		Y		Y		Y			N	Y	N		N	y			
19	y		Y		Y		Y			N		N		N	y			
20	y		Y		Y		Y			N		N		N	y			
21	y		Y		Y		Y			N		N		N	y			
22	y		Y		Y		Y			N		N		N	y			
23	y		Y		Y		Y			N		N		N	y			
24	y		Y		Y		Y			N		N		N	Y			
25	y		Y		Y		Y			N		N		N	Y			
26	y		Y		Y			N		N		N		N	Y			
27	y		Y		Y			N		N		N		N	Y			
28	y		Y		Y			N		N		N		N	Y			

29	y		Y		Y			N		N		N		N	y			
30	y		Y		Y			N		N		N		N	y			
31	y		Y		Y			N		N		N		N	y			
32	y		Y		Y			N		N		N		N	y			
33	y		Y		Y			N		N		N		N	y			
34	y		Y		Y			N		N		N		N	y			
35	y		Y		Y			N		N		N		N	y			
36	y		Y		Y			N		N		N		N	y			
37	y		Y		Y		Y			N		N		N	y			
38	y		Y		Y		Y			N		N		N	y			
39	y		Y		Y		Y			N		N		N	y			
40	y		Y		Y		Y			N		N		N	y			
41	y		Y		Y		Y			N		N		N	y			
42	y		Y		Y		Y			N		N		N	y			
43	y		Y		Y		Y			N		N		N	y			
44	y		Y		Y		Y			N		N		N	y			

45	y		Y		Y		Y			N		N		N	y			
46	y		Y		Y		Y			N		N		N	y			
47	y		Y		Y		Y			N		N		N	y			
48	y		Y		Y		Y			N		N		N	y			
49	y		Y		Y		Y			N		N		N	y			
50	y		Y		Y		Y			N		N		N	y			
51	y		Y	N	Y		Y			N		N		N	y			
52	y		Y	N	Y		Y			N		N		N	y			
53	y		Y	N	Y		Y			N		N		N	y			
54	y		Y	N	Y		Y			N		N		N	y			
55	y		Y	N	Y		Y			N		N		N	y			
56	y		Y	N	Y		Y			N		N		N	y			
57	y		y	N		N		N		N					Y			
58	Y		Y			N		N		N		N				Pangira		

59		N	Y			N		N		N		N		N		O<br>ki<br>di	Deep<br>burn	
60	Y			N		N		N		N		N		N			Burn	
61	Y		Y		Y		Y			N		N		N			Wast<br>ed	
62		N	Y			N		N	Y			N		N				
63	Y		Y			N		N		N		N	Y			N	Kitgu<br>m<br>boys	Su<br>pe<br>rfi<br>ci<br>al<br>bu<br>rn
64	Y		Y			N		N		N		N						
65	Y		Y			N		N		N		N	Y					
66	Y		Y			N		N		N		N			Y			
67	Y		Y			N		N		N		N			Y			

Compiled by .... Title .... Date .... Sign ....

Verified by .... Title .... Date .... Sign ....
