# Supplementary material for: Modelling optimal allocation of resources in the context of an incurable disease
Source: PLoS One. 2017 Mar 13;12(3):e0172401. doi: 10.1371/journal.pone.0172401 (PMC5347997; doi:10.1371/journal.pone.0172401)
Supplement: S2 File — (PDF) [file pone.0172401.s006.pdf]

PROGRAM FOR NODDING SYNDROME OUTREACH SERVICES FOR THE MONTH OF JULY 2012

Date	Activity Package	Requirements
	Screening	Weighing scales
	Treatment & Referals	Muac tapes
	Lab tests, Skin sniffs	Height boards
	Nutrition Assessment	Plumpy Nuts
	Supplimentary feeds	ATMIT
	Psychosocial Support	Lab reagents
	Health Education	Sodium Valproate
	Follow up Rx Outcome	Vitamin A
	Surveillance	Vitamin B6
		Vitamin B12
		Folic Acid
		Case forms
		Follow up forms
		N/S OPD register
		N/S Line-lists
		**Mobilisers:**
		LCIII
		Gisu
		i/c Health Units
		HA/HI
		LCI
		Schools
		VHTs
		**Time:**
		Starting: 10:00am
		Ending : 04:00pm

