# Supplementary material for: Modelling optimal allocation of resources in the context of an incurable disease
Source: PLoS One. 2017 Mar 13;12(3):e0172401. doi: 10.1371/journal.pone.0172401 (PMC5347997; doi:10.1371/journal.pone.0172401)
Supplement: S3 File — (PDF) [file pone.0172401.s007.pdf]

SN	IN SCHOOL	NEVER ATT	DROPPE	COMMENTS
1	yes			Received treatment once and did not come back
2	yes			Started treatment and dropped out
3	yes			stable but defaulted in September,
4		yes		He has been recieveing treatment consistently
5	yes			Taking his drugs consistently
6	yes			Taking drugs consistently and improving
7		Yes		Improving and adhering to treatment
8		Yes		Has dropped out of treatment
9	yes			Initiated on treatment but defaulted
10				Stable on medication but transferred to Gulu
11	yes			Stable on medication
12		yes		Started treatment and dropped out
13	yes			Started treatment and dropped out
14	yes			After review noted was epileptic
15			yes	Not regular in accessing treatment
16			yes	Stable on Medication
17		yes		Transferred to kitgum in September
18			yes	Impoved greatly and adhering to treatment
19	yes			Stable and adhering to medication
20	yes			Stable on medication
21	yes			Stable on medication
22			yes	Stable on medication with some improvement
23			yes	Stable on medication but defaulted
24			yes	Stable on medication
25			yes	Stable on medication
26	yes			Stable and on medication
27	yes			Stable and on medication
28			yes	Stable and on medication
29	yes			Stable and on medication
30	yes			Defaulted in October
31			yes	Stable and on medication
32	yes			Not regular in accessing treatment
33			yes	Defaulted
34			yes	Not regular in accessing treatment
35			yes	On medication with little improvement
36			yes	Stable and on medication
37	yes			Not regular in accessing treatment
38			yes	On medication, experiencing frequent attacks lately
39	yes			defaulted
40	yes			On medication
41			Yes	Lost to follow up
42			yes	Lost to follow up
43			Yes	Started treatment and defaulted in November
44			yes	Stable and on Medication
45		yes		Started treatment and droped out

46	yes			Started treatment and droped out
47			yes	Stable on medication but defaulted
48			yes	Stable and on Medication
49			yes	Stable and on Medication
50			yes	Stable and on Medication
51	yes			ON medication but not regularly collecitng drugs
52	yes			Stable and on Medication
52	yes			Stable and on Medication
54	yes			Stable and on Medication
55	yes			Stable on medication
56	yes			Stable and on Medication
57			yes	Stable and on Medication
58				Stable on treatment
59			yes	Stable and on Medication but defaulted in Novemeber
60			yes	Stable and on Medication
61		yes		Stable on medication
62		yes		Stable and on medication
63			yes	Stable and on medication
64			yes	Stable and on medication
65	yes			Started treatment defaulted in August
66	yes			Adhering to medication and improving
67	yes			Not regular in accessing treatment
68			yes	Adhering to medication and improving
69			yes	Stable and on medication
70	yes			Adhering to medication and improving
71			yes	Adhering to medication with slight improvement
72	yes			Adhering to treatment and improving
73			yes	Adhering to medication with slight improvement
74			yes	Stable and adhering to medication
75	yes			Adhering to medication with slight improvement
76	yes			Adhering to medication and improving
77			yes	Adhering to treatment with attacks only when it is cold
78			yes	Adhering to treatment with slight improvement
79			yes	Adhering with no improvement
80			yes	Adhering to medication with improvement
81		yes		Adhering to medication and improving
82			yes	Adhering to medication with improvement
83			yes	Adhering to medication with slight improvement
84	yes			Adhering to medication with improvement
85			yes	Adhering to medication with improvement
86	yes			Adhering to medication with little improvement
87			yes	Adhering to medication with slight improvement
88		yes		Adhering to medication with improvement
89			yes	Adhering to medication with improvement
90	yes			Adhering to medication with improvement
91			yes	Adhering to medication with improvement
92			yes	Adhering to medication with slight improvement

93	yes		Adhering to medication with slight improvement
94		yes	Adhering to medication with improvement
95	yes		Adhering to medication with improvement
96	yes		Adhering to medication with slight improvement
97		yes	Adhering to medication and is stable
98	yes		Adhering to medication with improvement
99	yes		Adhering to medication with improvement
100	no	yes	Received treatment once and did not come back
101	yes	no	Improving and adhering to treatment
102	yes		He has been recieveing treatment consistently
103	yes		Taking his drugs consistently
104	yes		Taking drugs consistently and improving
105	no		Improving and adhering to treatment
106	no	yes	Adeharing to medication and resume school
107	no	yes	Improving and adhering to treatment
108	yes		Improved and adhering to treatment
109	yes		Improved and adhering to treatment
110	no	yes	Impoved greatly and adhering to treatment
111		yes	Stable and adhering to medication
112		yes	Stable on medication
113		yes	Stable on medication
114		yes	Improved but detorate
115		yes	Improved and adhering to treatment
116	yes		Improved and adhering to treatment
117		yes	Some Improvement
118		yes	Improved and adhering to treatment
119		yes	Improved and adhering to treatment
120		yes	Improved and adhering to treatment
121		yes	Improved and adhering to treatment
122		yes	Marked Improvement and adhering to treatment
123		yes	Marked Improvement and adhering to treatment
124		yes	Marked Improvement and adhering to treatment
125		yes	Some improvement
126		yes	Marked Improvement and adhering to treatment
127	yes		Marked Improvement and adhering to treatment
128	yes		Marked Improvement and adhering to treatment
129		yes	Marked Improvement and adhering to treatment
130		yes	Improvement and adhering to treatment
131	yes		Improvement and adhering to treatment
132	yes		Improvement and adhering to treatment
133		yes	Adhering to medication with no improvement
134		yes	Adhering to medication with improvement
135		yes	Adhering to medication with improvement
136	yes		Adhering to medication with improvement
137	yes		Adhering to medication with improvement
138	yes		Adhering to medication with improvement
139	yes		was stable on medication but defaulted in August

140		yes		Adhering to medication with improvement
141	yes			Adhering to medication with slight improvement
142	yes			Adhering to medication with improvement
143	yes			Adhering to medication with improvement
144			yes	Adhering to medication with improvement
145			yes	Adhering to medication with improvement
146	yes			Adhering to medication with little improvement
147	yes			Adhering to medication with improvement
148			yes	Adhering to medication with improvement
149	yes			Adhering to medication with improvement
150			yes	Adhering to medication with improvement
151	yes			Adhering to medication with improvement
152	yes			Adhering to medication with improvement
153			yes	Adhering to medication with improvement
154			yes	Adhering to medication with slight improvement
155	yes			Adhering to medication with improvement
156			yes	not regular in collecting her drugs
157			yes	Adhering to medication with improvement
158			yes	Adhering to medication with improvement
159			yes	Adhering to medication with improvement
160	yes			Adhering to medication with improvement
161	yes			Adhering to medication with improvement
162			yes	Adhering to medication with improvement
163		yes		Adhering to medication with improvement
164			yes	Adhering to medication with improvement
165			yes	Adhering to medication with much improvement
166			yes	Adhering to medication with slight improvement
167	yes			Adhering to medication with slight improvement
168			yes	Adhering to medication with slight improvement
169	yes			Adhering to medication with improvement
170	yes			Adhering to medication with improvement
171			yes	Adhering to medication with slight improvement
172			yes	Adhering to medication with improvement
173	yes			Adhering to medication with improvement
174	yes			Adhering to medication with improvement
175	yes			Adhering to medication with improvement
176			yes	Adhering to medication with much improvement
177	yes			Adhering to medication with much improvement
178			yes	Adhering to medication with improvement
179	yes			Adhering to medication with improvement
180			yes	not regular in collecting her drugs
181	yes			Adhering to medication with improvement
182			yes	Adhering to medication with slight improvement
183	yes			Adhering to medication with improvement
184			yes	Adhering to medication with improvement
185	yes			Adhering to medication with improvement
186	yes			Adhering to medication with improvement

187			yes	Adhering to medication with improvement
188			yes	Adhering to medication with improvement
189			yes	Defaulted but is back with many episodes od seizures
190	yes			Adhering to medication with improvement
191	yes			Adhering to medication with improvement
192	yes			Adhering to medication with improvement
197	yes			Adhering to medication with slight improvement
194			Yes	adhering to treatment with no improvement
195			yes	Adhering to medication with slight improvement
196	yes			Adhering to medication with slight improvement
197			yes	Adhering to medication with slight improvement
198			yes	Adhering to medication with improvement
199	yes			Adhering to medication with improvement
200			yes	Adhering to medication with marked improvement
201			yes	Adhering to medication with improvement
202			yes	Adhering to medication with marked improvement
203			yes	Adhering to medication with slight improvement
204			yes	Adhering to medication with slight improvement
205	yes			Stable on medication and dropped out in Oct
206	yes			Defaulted
207		yes		Stable on medication but defaulted in December
208			yes	Stable on medication but defaulted in December
209		yes		Reviews indicated the patient had epilepsy
210	yes			Adhering to medication with improvement
211	yes			Adhering to medication with improvement
212	yes			Stable on Medication with slight improvement
213	yes			Adhering to medication with slight improvement
214	yes			Adhering to medication with slight improvement
215	Resumed School			Adhering to medication with slight improvement
216	yes			Adhering to medication with improvement
217			yes	Adhering to medication with slight improvement
218	yes			Adhering to medication with litt;e improvement
219			yes	Adhering to medication with improvement
220			yes	Adhering to medication with improvement
221	yes			Adhering to medication with marked improvement
222		yes		Adhering to medication with marked improvement
223			yes	Adhering to medication with improvement
224			yes	Improvement and adhering to treatment
225			yes	Marked Improvement and adhering to treatment
226			Yes	Some Improvement
227			yes	Marked Improvement and adhering to treatment
228			yes	Some Improvement
229			yes	Improvement and adhering to treatment
230	Yes			Marked Improvement and adhering to treatment
231	Yes			Some Improvement
232			yes	Some Improvement
233	Yes			Improvement and adhering to treatment

234				yes			Some Improvement
235						yes	Marked Improvement and adhering to treatment
236		Yes					Marked Improvement and adhering to treatment
237						yes	Marked Improvement and adhering to treatment
238		Yes					Marked Improvement and adhering to treatment
239		Yes					Some Improvement
240						yes	Improvement and adhering to treatment
241				yes			Some Improvement
242					yes		Adhering to medication with slight improvement
243					yes		Adhering to medication with marked improvement
244					yes		Adhering to medication with slight improvement
245							Adhering to medication with improvement
246					yes		Adhering to medication with improvement
247			yes				Adhering to medication with improvement
248	yes						Adhering to medication with slight improvement
249					yes		Adhering to medication with marked improvement
250	yes						Adhering to medication with improvement
251					yes		Little Improvement while adhering to medication
252						yes	Improvedment and adhering to medication
253		yes					Improvedment and adhering to medication
254						yes	Improvedment and adhering to medication
255	yes						Adhering to medication with improvement
256					yes		Adhering to medication with improvement
257					yes		Adhering to medication with improvement
258	yes						Stable on medication but defaulted
259					yes		Accessing treatment but some times skips treatment
260					yes		Accessing treatment but some times skips treatment
261					yes		Adhering to medication with marked improvement
262					yes		Adhering to medication with slight improvement
263					yes		Adhering to medication with improvement
264	yes						Adhering to medication with no improvement
265	yes						Lost to follow up
266					yes		Accessed treatment for four month and dropped out in October
267	yes						defaulted in August
268					yes		After further review discovered was epileptic
269					yes		Adhering to medication with slight improvement
270					yes		Adhering to medication with improvement
271	yes						Adhering to medication with slight improvement
272					yes		Adhering to medication with slight improvement
273			yes				Adhering to medication with improvement
274	yes						Defaulted
275	yes						Adhering to medication with improvement
276	yes						Defaulted
277					yes		defaulted
278					yes		Adhering to medication with slight improvement
279					yes		Adhering to medication with improvement
280	yes						Adhering to medication with improvement

281					yes	Adhering to medication with improvement
282					yes	Adhering to medication with improvement
283					yes	Adhering to medication with improvement
284					yes	Adhering to medication with improvement
286			yes			Adhering to medication with improvement
287			yes			Adhering to medication with improvement
288					yes	Adhering to medication with improvement
289	yes					Adhering to medication with improvement
290	yes					Adhering to medication with improvement
291	yes					Adhering to medication with improvement
292				Yes		Marked Improvement
293		yes				Marked Improvement
294			yes			Adhering to medication with marked improvement
295	yes					Adhering to medication with marked improvement
295	yes					Adhering to medication with marked improvement but defaulted in
296	yes					Adhering to medication with marked improvement but defaulted in
297					yes	Reported for treatment and dropped out after three visits
298					yes	Reported to the clinic and dropped out after 2 treatment sessions
299	yes					Adhering to medication with marked improvement but defaulted in
300					yes	Accessed treatment with marked improvement but dropped out in
301	yes					Not regularly getting drugs
302	yes					Reported as a nodding case after further screening noted as epileps
303	yes					Not regularly getting drugs
304	yes					Defaulted
305	yes					Adhering to medication with improvement
306	yes					Not regularly accessing treatment but stable with medication
307					yes	Adhering to medication with marked improvement

December
December

December
December

y
