# Supplementary material for: Modelling optimal allocation of resources in the context of an incurable disease
Source: PLoS One. 2017 Mar 13;12(3):e0172401. doi: 10.1371/journal.pone.0172401 (PMC5347997; doi:10.1371/journal.pone.0172401)
Supplement: S4 File — (PDF) [file pone.0172401.s008.pdf]

ID No	PARISH	SUBCOU	AGE	SEX	DEC	JAN	FEB	MAR
			15	F				
			14	F				
			14	F				
			13	M				
			12	F				
			12	M				
			13	M			R	
			9	M				
			14	M				
			14	M				
			4	M				
			7	M				
			16	M				
			18	M				

APL	MAY
R	R
