# Supplementary material for: Modelling optimal allocation of resources in the context of an incurable disease
Source: PLoS One. 2017 Mar 13;12(3):e0172401. doi: 10.1371/journal.pone.0172401 (PMC5347997; doi:10.1371/journal.pone.0172401)
Supplement: S5 File — (PDF) [file pone.0172401.s009.pdf]

LINE LIST FOR NODDING DISEASE ATTENDING OPD SERVICES IN KITGUM (

S/NO	SEX	WEIGHT	NEW ATT	RE-ATT	DIAGNOSIS	DIAGNOSIS
	M	52Kg	y		Epilepsy	
	M	37Kg	y			Nod syn PLUS
	M	42kg	y			Nod syn PLUS
	F	30kg	y			Epilepsy
	M	34Kg	y			Nod syn PLUS
	F	22Kg	y		Epilepsy	
	M	41.5Kg	y			Nod syn PLUS
	F	38.9Kg	y			Nod syn PLUS
	F	43.7Kg	y			Nod syn PLUS
	M	33Kg	y			Nod syn PLUS
	M	34Kg	y			Nod Syndrom
	m	44Kg	y		Epilepsy	
Sub Total						
13	M	18Kg	y		Epilepsy	Nod syndrom
14	M	53Kg	y			Nod Syndrom
15	F	37Kg	y			Nod Syndrom
16	M	12Kg	y		Epilepsy	
17	M	50Kg	y		Epilepsy+Others	
18	M	35Kg	y			Head Nodd Syn
19	M	35Kg	y		Epilepsy	
20	M	46Kg	y			Nod syn PLUS
21	F	25Kg	y			Nod Syn
22	F	31Kg	Y			Nod syn PLUS
23	F	26Kg	y			Nod Syn
24	F	35Kg	y			Nod Syn
25	M	25Kg	y			Nod syn PLUS
26	F	32Kg	y			Nod Syn
27	F	27Kg	y			Nod syn PLUS
Sub Total						
28	F	33.5Kg	y			Nod Syn Plus
29	M	34.1Kg	y			Nod Syn Plus
30	M	25.7Kg	y			Nod Syn Plus
31	F	43.5Kg	y			Nod Syn
32	F	28.8Kg	y		Epilepsy	
33	M	27.5Kg	y			Nod Syn Plus
34	F	39.3Kg	y			Nod Syn Plus
35	M	39.5Kg	y			Nod Syn
36	M	43.5Kg	y			Nod Syn Plus
37	M	43Kg	y			Nod Syn Plus
38	M	34.8Kg	y		Epilepsy	
39	M	22.5Kg	y		Epilepsy	

Sub Total						
	40	F	25.1Kg	y		Nod Syndrom
	41	F		y		Nod Syn Plus
	42	F	36.2Kg	y		Nod Syn Plus
	43	M		y	Epilepsy	
	44	M	5.1Kg	y	Epilepsy	
	45	M	28.3Kg	y		Nod Syn Plus
	46	M	30.7Kg	y		Nod Syn Plus
	47	F	???	y		
	48	M	30.3Kg	y		Nod Syn Plus
	49	M	34.5Kg	y		Nod Syn Plus
	50	M	17.5Kg	y		Nod Syn
	51	F	13.4Kg	y		Nod Syn Plus
	52	F	29.6Kg	y		Nod Syn Plus
	53	M	45Kg	y		Nod Syn Plus
	54	M	30.5Kg	y		Nod Syn
	55	F	25Kg	y		Nod Syn Plus
	56	M	53Kg	y		Nod Syn Plus
Sub Total						
	57	F	34.6Kg	y		Head Nod Syn
	58	M	51.6Kg	y		Nod Syn Plus
	59	M	52.3Kg	y		Nod Syn Plus
	60	F	37.6Kg	y		Nod Syn Plus
	61	F	30.2Kg	y		Nod Syn Plus
	62	F	34Kg	y		Nod Syn Plus
	63	M	26.4Kg	y		Nod Syn
	64	F	24.5Kg	y		Nod Syn
Sub Total						
	65	M	???	y		Nod Syn Plus
	66	M		y		Nod Syn Plus
	67	M	33Kg	y		Nod Syn Plus
	68	M	34Kg	y		Nod Syn Plus
	69	F	???	y	Epilepsy	
	70	M	31Kg	y		Nod Syn Plus
	71	F	???	y		NOD Syn
	72	M	???	y		Nod Syn Plus
	73	F	???	y		Nod Syn Plus
	74	M	???	y		Nod Syn
	75	F	???	y		Nod Syn
	76	M	???	y		Nod Syn Plus
	77	F	???	y		Nod Syn Plus
	78	F	???	y		Nod Syn Plus
	79	F	???	y		Nod Syn Plus
	80	F	???	y		Nod Syn Plus

	81 F	???	y		Nod Synd
	82 M	???	y		Nod Syn Plus
	83 M	???	y		Nod Synd
	84 F	???	y	Epilepsy	
	85 F	???	y		Nod Syn
	86 M	???	y		Nod Syn Plus
	87 M	???	y		Nod Syn
	88 M	???	y		Nod Syn Plus
	89 M	???	y		Nod Syn Plus
	90 F	???	y		Nod Syn Plus
	91 M	???	y		Nod Syn Plus
	92 M	???	y		Nod Syn
	93 F	???	y	Epilepsy	
	94 M	???	y		Nod Syn
	95 M	???	y		Nod Syn
	96 M	???	y		NodPlus
	97 M	???	y		NodPlus
	98 F	???	y		NodPlus
	99 F	???	y		NodPlus
Sub Total					
	100 F	???	y		Nodiing Plus
	101 M	???	y		Nodiing Plus
	102 M	???	y		Nodiing Plus
	103 M	???	y		Nodiing Plus
	104 F	???	y		Nodiing Plus
	105 M	???	y		Nodiing Plus
	106 M	???	y		Nodiing Plus
	107 F	???	y		Nodiing Plus
	108 M	???	y		Nodiing Plus
	109 M	???	y		Nodiing Plus
	110 M	???	y		Nodiing Plus
	111 M	???	y	Epilepsy	
	112 M	???	y		Nod SYN
	113 M	???	y		Nod SYN
	114 F	???	y		Nod SYN
	115 F	???	y		Nod Pplus
	116 F	???	y		Nod Syn
	117 M	???	y	Epilepsy	
	118 M	???	y		Nod SYN
	119 M	???	y		Nod PLUS
	120 F	???	y		Nod Plus
	121 F	???	y		Nod PLUS
	122 F	???	y	Epilepsy	Nod PLUS
	123 F	???	y		Nod PLUS
	124 M	???	y		Nod PLUS
Sub Total					

	125	M		y		Nod PLUS
	126	M	24.5Kg	y		Nod PLUS
	127	M		y		Nod PLUS
	128	M	41.9Kg	y		Nod PLUS
	129	M		y		Nod PLUS
	130	M	44.9Kg	y		Nod PLUS
	131	M	25.2Kg	y		Nod PLUS
	132	F	37.1kg	y		Nod PLUS
	133	F	41.6Kg	y		Nod PLUS
	134	F	36.5Kg	y		Nod PLUS
Sub Total						
	135	F	28.8Kg	y		Nod PLUS
	136	F	33Kg	y		Nod PLUS
	137	F		y		Nod PLUS
	138	M	44Kg	y	Epilepsy	
	139	F	28.8Kg	y		Nod PLUS
	140	F	50.8Kg	y		Nod PLUS
	141	F	43.5Kg	y		Nod PLUS
	142	F	33.3Kg	y		Nod PLUS
	143	F	21.3Kg	y	Epilepsy	
	144	F	38.5Kg	y		Nod PLUS
	145	M	36Kg	y		Nod PLUS
	146	F	52.6Kg	y		Nod PLUS
	147	F	41.6Kg	y		Nod PLUS
	148	F	46.6Kg	y		Nod SYN
	149	F	43.6Kg	y		Nod SYN
	150	M	48.5Kg	y	Epilepsy	
	151	M	38.1Kg	y		Nod PLUS
	152	F	27.4Kg	y		Nod Syn
	153	M	43.2Kg	y	Epilepsy	
	154	F		y		Nod Syn
	155	F	37.2Kg	y		Nod PLUS
Sub Total						
	156	F	25.3Kg	y		Nod Synd
	157	M	30.2kg	y	Epilepsy	
	158	F	30Kg	y	Epilepsy	
	159	M	24.2Kg	y		Nod Synd
	160	F	47.3Kg	y	Epilepsy	
	161	F	60Kg	y	Epilepsy	
	162	m	59.4Kg	y	Epilepsy	
	163	F	55Kg	y		Nod Synd plus
	164	F	52Kg	y		other seizures
	164	M	47Kg	y	Epilepsy	
	165	M	48Kg	y	Epilepsy	

	166	M	31.8Kg	y			Head Nodding
	167	M	43.6Kg	y		Epilepsy	
	168	F	38.7Kg	y			Head Nodding
	169	F	36.4Kg	y		Epilepsy	
	170	M	36.1Kg	y			Head Nodding
	171	F	24.9Kg	y			Head Shaking
	172	M	32.7Kg	y			Head Nodding
	173	M	28.2Kg			Epilepsy	
	174	M	28.5Kg		Y		Nod Syn
	175	F			y	Epilepsy	
Sub Total							
	176	F	22.7Kg	y			Ns Plus
	177	F	37Kg		y		Ns Plus
	178	M	42.3Kg		y		Ns Plus
	179	F	53.6Kg	y		Epilepsy	
	180	M	46.8Kg		y		Ns Plus
	181	M	47.1Kg		y	Epilepsy	
	182	M	44.5Kg		y		Ns Plus
	183	M	43Kg		y		Ns Plus
	184	M	42Kg		y		
	185	M	34.8Kg				NS
	186	M	36.6Kg	y			Ns Plus
	187	M	35.6Kg	y			Ns Plus
	188	F	16.2Kg	y			Ns Plus
	189	F	27Kg		y		Ns Plus
	190	F	38.8Kg		y		Ns Plus
	191	M	21Kg		y		Ns Plus
	192	M	28.2Kg	y			NS
	193	F	46.6Kg	y		Epilepsy	
	194	F		y			NS Plus
	195	M		y		Epilepsy	
Sub Total							
	196	f	37.4Kg	y			NS Plus
	197	M	56.3Kg	y			NS Plus
	198	M	28.8Kg	y		Epilepsy	
	199	M	33.6Kg	y			NS Plus
	200	M	24.6Kg	y			NS Plus
Sub Total							
	201	F	38.5Kg				
	202	F	23.4Kg				NS Plu
	203	M	37.6Kg	y			PEM
	204	M	32.6Kg	y			Nod SYND
	205	M	19.6Kg	y			
Sub Total							

	206	M	33.4Kg	y			Nod SYND Plus
	207	M	35.8Kg	y			Nod SYND Plus
	208	M	37.9Kg	y			Nod SYND Plus
	209	F	23.1Kg	y			Nod SYND Plus
	210	M	20.1Kg	y			Nod SYND Plus
	211	F	30.0Kg	y			Nod SYND Plus
	212	M	19.7Kg	y			Nod SYND Plus
	213	F	40.5Kg	y			Nod SYND Plus
	214	F	40Kg	y			Nod SYND Plus
	215	F	23.7Kg	y			Nod SYND Plus
	216	M	54.3Kg	y			Nod SYND Plus
	217	F	44.5Kg	y			Nod SYND Plus
	218	F	37.9Kg	y			Nod SYND Plus
	219	M	31.6Kg	y			Nod SYND Plus
	220	F	37.6Kg	y			Nod SYND Plus
	221	M	35.1Kg	y			Nod SYND Plus
	222	F	44.4Kg	y			Nod SYND Plus
	223	M	52.3Kg				Nod SYND Plus
	224	M	40Kg	y		Epilepsy	
	225	m	22.2Kg	y		Epilepsy	
Sub Total							
	226	M	45.8Kg	y			Ns Plus
	227	M	35.9Kg		y		NS Plus
	228	F	54.1Kg		y	Epilepsy	
	229	F	35.6Kg		y		Nod Syn
	230	F	32Kg		y		NS pLUS
	231	M	31.4Kg		y		NS Plus
	232	F	45Kg	y			NS Plus
	233	M	51Kg	y			NS
	234	M	41Kg	y			NS Plus
	235	M	25.9Kg	y			NS Plus
	236	F	34Kg	y			NS pLUS
	237	F	28.8Kg	y			NS Plus
	238	F	29.2Kg	y			NS
Sub Total							
	239	M	44.9Kg		y		NS
	240	F	25.5Kg	y			NS
	241	M	30.8Kg	y			NS Plus
	242	M	36Kg		y		NS Plus
	243	M	32.7Kg	y			NS
	244	F	30.3Kg	y			NS Plus
	245	M	33Kg	y			NS Plus
	246	M	26Kg		y		NS Plus
	247	F	37Kg	y			NS

	248	M	16.1Kg	y			Head NS
Sub Total							
	249	F	43.5Kg		y		Head NS
	250	F	42Kg	y			Head NS Plus
	251	M	34.5Kg	y			Head NS
	252	F	29.1Kg	y			Head NS Plus
	253	M	54.6Kg	y			Head NS Plus
	254	M	26.2Kg	y			Head NS
	255	F	42Kg	y			Head NS Plus
Sub Total							
	256	F	31Kg	y		Epelipsy	
	257	F	30Kg	y			NS plus
	258	F	29.3Kg	y			NS plus
	259	F	45.8Kg	y			Head NS
Sub Total							
	260	M	34Kg		y		NS Plus
	261	M	22.8Kg	y			NS Plus
	262	M	42.1Kg	y		Epilepsy	
	263	M	28.7Kg	y			NS pLUS
	264	F	42.2Kg	y			NS Plus
Sub Total							
	265	M	32.9Kg	y			NS Plus
	266	F	58.8Kg	y			NS Plus
	267	M	9.6Kg	y		Epilepsh	
Sub Total							
	268	F			Y		NS Plus
	269	M	32.2Kg		Y		NS
	270	M	51Kg	Y			NS Plus
	271	M	45.5Kg	Y			NS Plus
Sub Total							
	272	F	45.6Kg	y			NS Plus
	273	M	36.8Kg	y			NS
	274	M	38.1Kg	y			NS
	275	M	32.7Kg	y		Epilepsy	
Sub Total							
	276	F	50.3Kg	Y			NS
	277	M	11Kg	Y		Epilepsy	
Sub Total							
	278	M	26.2Kg	y			

	279	F		y		
	280	M	9.1Kg			
	281	F	18Kg	y		
	282	F	45.8Kg	y		
	283	M	36Kg	y		
	284	M	23.3Kg	y		
	285	F	35.1Kg	y		
	286	M		y		
	287	F	38.3Kg	y		
	288	M	20Kg	y		
	289	F	31.7Kg	y		
	290	M		y		
	291	F		y		
	55	F	38Kg		y	NS Plus
	91	M	58.2Kg		y	NS Plus
	292	M		y		Head Nodding
	293	F		y		Head Nodding
	294	M	21.8Kg	y		Head Nodding
	295	M	52.3Kg	y		Head Nodding
	194	M	37Kg	y		Head Nodding
Sub Total						
	14	M				
	181	F				
	117	F				
	29	M				
	246	F				
	39	M				
	255	M				
	250	M				
	2	M				
	289	M				
Sub Total						
	21	F				
	296	M				
	16	M				
	37	M				
	296	M				
	101	M				
	297	F				
Sub Total						
	298	M				
	299	F				
	10	M				
	9	F				

	300	M
	58	M
	132	M
	242	F
	122	F
	173	
	164	
	138	
	301	
	158	
	302	
Sub Total		
	262	
	148	
	166	
	110	
	157	
	14	
	50	
	142	
	90	
	154	
	303	
	259	
	159	
	51	
	247	
	204	
	62	
	133	
	116	
	8	
	48	
	308	
Sub Total		
	292	
	61	
Sub Total		
	272	
	260	
	266	
	47	
	56	
	101	

267
268
201
42
191
46
68
309
283

GOVERNMENT HOSPITAL:

DRUGS/TREATMENT	REF-IN	REF-OUT
CB2,ALB,Multivite,Vit B Comp		
SV,VIT A, ALB,VIT B12		
SV,VIT A, ALB,VIT B12		
CBZ, F/A		
SV,VIT A, ALB,VIT B12		
CBZ, F/A		
SV,VIT A, ALB,VIT B12		
SV,VIT A, ALB,VIT B12		
SV,VIT A, ALB,VIT B12		
SV,VIT A, ALB,VIT B13		
Sodium Valporate ,Alb+Vit A		
CB2,ALB,Multivite.		
SV,VIT A, ALB,VIT B12		
Sodium Valporate ,Alb+Vit A		
Sodium Valporate ,Alb+Vit A		
Vit A, Vit B12,Alb SV		
SV,VIT A, ALB,VIT B12		
SV,VIT A, ALB,VIT B12		
CB2,ALB,Vit A.		
SV,VIT A, ALB,VIT B12		
SV,VIT A, ALB,VIT B12		
SV,VIT A, ALB,VIT B12		
SV,VIT A, ALB,VIT B12		
SV,VIT A, ALB,VIT B12		
SV,VIT A, ALB,VIT B12		
Sodium Valporate ,Alb		
Sodium Valporate ,Alb+Vit A		
SV,VitB complex,Vit A,Alb, Multivite		
SV,VitB complex,Vit A,Alb, Multivite		
SV,VitB complex,Vit A,Alb, Multivite		
SV,VitB complex,Vit A,Alb, Multivite		
SV,VitB complex,Vit A,Alb, Multivite		
SV,VitB complex,Vit A,Alb, Multivite		
SV,VitB complex,Vit A,Alb, Multivite		
SV,VitB complex,Vit A,Alb, Multivite		
SV,VitB complex,Vit A,Alb, Multivite		
CPZ ,Folic Acid,Alb		
CPZ Folic Acid,Alb		
Alb, Vit A,Na valp,Vit B Complex		

Admit,Na Valp
VitA, Vit B Complex, Na Valp
VitA, Vit B Complex, Na Valp
Admit on CBZ,Vit A
CBZ
Na Valp, Vit Bcomp,Vit A, Alb
Na Valp, Vit Bcomp,Vit A, Alb
Nutitional Therapy
Na Valp, Vit Bcomp,Vit A, Alb
Na Valp, Vit Bcomp,Vit A, Alb
Na Valp, Vit Bcomp,Vit A, Alb
Na Valp, Vit Bcomp,Vit A, Alb
Na Valp, Vit Bcomp,Vit A, Alb
Na Valp, Vit Bcomp,Vit A, Alb
Na Valp, Vit Bcomp,Vit A, Alb
Na Valp, Vit Bcomp,Vit A, Alb
Na Valp, Vit Bcomp,Vit A, Alb

Na Valp,Vit A, Vit BC,Multivite,Alb
Na Valp,Vit A, Vit BC,Multivite,Alb
Na Valp,Vit A, Vit BC,Multivite,Alb
Na Valp,Vit A, Vit BC,Multivite,Alb
Na Valp,Vit A, Vit BC,Multivite,Alb
Na Valp,Vit A, Vit BC,Multivite,Alb
Na Valp,Vit A, Vit BC,Multivite,Alb
Na Valp,Vit A, Vit BC,Multivite,Alb

Na Valp,Multivite,Vit B Comp,Vita A, Alb,Cloxa
Na Valp,Multivite,Vit B Comp,Vita A, Alb,
Na Valp,Multivite,Vit B Comp,Vita A, Alb,
Na Valp,Multivite,Vit B Comp,Vita A, Alb,
CBZ Vit B Complex
Na Valp, Vit B Comp,Vit A, Alb.
Na Valp, F/A,Alb, Vit A.
Na Valp, F/A.
Na Valp, F/A
Na Valp,F/A,Alb,Vit A
Na Valp, F/A,Alb, Vit A.
Na Valp, Vit B Comp,Vit A, Alb.
Na Valp, Vit B Comp,Vit A, Alb.
Na Valp, Vit B Comp,Vit A, Alb.
Na Valp, Vit B Comp,Vit A, Alb.
Na Valp, Vit B Comp,Vit A, Alb.

NaValp,Vit B Comp, VitA Alb.
Na Valp, Vit B Comp,Vit A, Alb.
NaValp,Vit B Comp, VitA Alb.
Na Valp,Vit A,Vit B Comp,Multivite
Na Valp,Alb,Vit B Comp,Vit A.
Na Valp, Vit B Comp,Vit A, Alb.
Na Valp, Vit B Comp,Vit A, Alb.
Na Valp, Vit B Comp,Vit A, Alb.
Na Valp, Vit B Comp,Vit A, Alb.
Na Valp, Vit B Comp,Vit A, Alb.
Na Valp, Vit B Comp,Vit A, Alb.
Na Valp, Vit B Comp,Vit A, Alb.
Na Valp, Vit A, Muitivite,Vit b Comp,Alb
Na Valp, Vit A, Muitivite,Vit b Comp,Alb
Na Valp, Vit A, Muitivite,Vit b Comp,Alb
Na Valp,Vit A,Vit B Comp,Alb
Na Valp,Vit A,Vit B Comp,Alb
Na Valp,Vit A,Vit B,Alb.Diazpam iv
Na Valp,Vit A,Vit B,Alb.

Na Valp,Vit A,Vit B, Multvit,Alb
Na Valp,Vit A,Vit B, Multvit,Alb
Na Valp,Vit A,Vit B, Multvit,Alb
Na Valp,Vit A,Vit B, Multvit,Alb
Na Valp,Vit A,Vit B, Multvit,Alb
Na Valp,Vit A,Vit B, Multvit,Alb
Na Valp,Vit A,Vit B, Multvit,Alb
Na Valp,Vit A,Vit B, Multvit,Alb
Na Valp,Vit A,Vit B, Multvit,Alb
Na Valp,Vit A,Vit B, Multvit,Alb
Na Valp,Vit A,Vit B, Multvit,Alb
Na Valp Vit B Comp Muilltivit, Alb
Na Valp Vit B Comp Muilltivit, Alb
Na Valp Vit B Comp Muilltivit, Alb
Na Valp Vit B Comp Muilltivit, Alb
Na Valp Vit B Comp Muilltivit, Alb
Na Valp, Vit B Comp,Vit A, Alb
Na Valp, Vit B Comp,Vit A, Alb
Na Valp, Vit B Comp,Vit A, Alb
Vit B12,Alb,Vit A, Na Valp
Na Valp,Multivitt,Alb,Vit B12
Na Valp,Multivitt,Alb,Vit B12
Na Valp,Multivitt,Alb,Vit B12
Na Valp,Multivitt,Alb,Vit B12
Na Valp,Multivitt,Alb,Vit B12

Na Valp,Alb,Vit A
Na Valp,Alb,Vit A
Na Valp,Alb,Vit A
Na Valp,Vit B Comp,Alb,Vit A.
Na Valp,Vit B Comp,Alb,Vit A.
Na Valp,Vit B Comp,Alb,Vit A.
Na Valp,Vit B Comp,Alb,Vit A.
Na Valp,Vit B Comp,Alb,Vit A.
Na Valp,Vit B Comp,Alb,Vit A.
Na Valp,Vit B Comp,Alb,Vit A.

Na Valp,Vit B Comp,Alb,Vit A.
Na Valp,Vit B Comp,Alb,Vit A.
Na Valp,Vit B Comp,Alb,Vit A.
Na Valp,Vit B Comp,Alb,Vit A.
Na Valp,Vit B Comp,Alb,Vit A.
Na Valp,Vit B Comp,Alb,Vit A.
Na Valp,Vit B Comp,Alb,Vit A.
Na Valp,Vit B Comp,Alb,Vit A.
CBZ,Na Valp,Vit B Comp,Alb,Vit A.
Na Valp,Vit B Comp,Alb,Vit A.
Na Valp,Vit B Comp,Alb,Vit A.
Na Valp,Vit B Comp,Alb,Vit A.
Na Valp,Vit B Comp,Alb,Vit A.
Vit A,VitB12,Na Valp
Vit A,VitB12,Na Valp
CBZ,Phytoin,F/A
Na Valp,Vit B Comp,Alb,Vit A.
Na Valp,Vit B Comp,Alb,Vit A.
F/A, phentoin
Vit A,VitB12,Na Valp
Vit A,VitB12,Na Valp

Vit A,Vit B12,Na Valp,
NaValp, Vit Bcomp,Mvt,Alb
phenitpion,Mvt,F/A
phenitpion,Mvt,F/A
CBZ,Mvit, Vit Bcomp
Phenobabiton,Bcomp,Muvt
pheytain,F/A,mvt Vit B Comp
Na Valp,Vit B Comp,Mvt,Alb,Vit A
Na Valp,Vit B Comp,Mvt,Alb,Vit A
CBZ,Mvt,Vit Bcomp,Alb Vit A
CBZ,Mvt,Vit Bcomp,Alb Vit A

CBZ,Mvt,Vit Bcomp,Alb Vit A
Na Valp,Vit A, Multivite
CBZ, F/A
Na Valp,Vit B12 Alb,VitA,B
CBZ,multivit,Alb,Vit B Comp.
Na Valp,Vit B12
CBZ,F/A
NA valp, Vit B12
CBZ,F/A

Na Valp,Vit B Comp,Mulvt,Alb Admit
Na Valp,Vit B Comp,Mulvt,Alb
Na Valp,Vit B Comp,Mulvt,Alb
CBZ,F/A
Na Valp,Vit B Comp,Mulvt,Alb
CBZ,Mvt Vit B Comp
Na Valp,Vit B Comp,Muvt
Na Valp,Vit B Comp,Muvt

NaValp,VIT B12,Muvt,Vit A
NaValp,VIT B12,Muvt,Vit A
NaValp,VIT B12,Muvt,Vit A
NaValp,VIT B12,Muvt,Vit A
NaValp,VIT B12,Muvt,Vit A
NaValp,VIT B12,Muvt,Vit A
NaValp,VIT B12,Muvt,Vit A
Na Valp,VitB12,
CBZ,R/A
Vit A,Vit B12,Na Valp.
CBZ,Vit B admit

Na Valp,Alb,VIT A CBZ
Na Valp,Alb,CBZ,Vit A,Vit B12
Na Valp,CBZ,FA,Vit A, Vit B12
Na Valp,CBZ,FA,Vit A, Vit B12
Na Valp,B comp, Alb,Vit A,Nutrition

Na Valp,Alb,Vit B, Vit A

Na Valp,Alb,Vit B, Vit A

Na Valp,Vit B Comp,Alb
Na Valp,Vit B Comp,Alb Vit A
Na Valp,Vit B Comp,Alb Vit A
Na Valp,Vit B Comp,Alb Vit A
Na Valp,Vit B Comp,Alb Vit A
Na Valp,Vit B Comp,Alb Vit A
Na Valp,Vit B Comp,Alb Vit A
Na Valp,Vit B Comp,Alb Vit A
Na Valp,Vit B Comp,Alb Vit A
Na Valp,Vit B Comp,Alb Vit A
Na Valp,Vit B Comp,Alb Vit A
Na Valp,Vit B Comp,Alb Vit A
Na Valp,Vit B Comp,Alb Vit A
Na Valp,Vit B Comp,Alb Vit A
Na Valp,Vit B Comp,Alb Vit A
Na Valp,Vit B Comp,Alb Vit A
Na Valp,Vit B Comp,Alb Vit A
Na Valp,Vit B Comp,Alb Vit A
CBZ,F/A
CBZ,F/A,Multivite

Na Valp,Vit B Comp,Alb,Vit A.Mvt
Navalp,VitB12,Vit A,CBZ,Folic Acid
CBZ,FA,Vit B12
Na Valp, Vit B12, Vit A, Alb
NaValp,Vit B Comp, VitA Alb.
NaValp,Vit B Comp, VitA Alb.
NaValp,Vit B Comp, VitA Alb.
NaValp,Vit B Comp, VitA Alb.
NaValp,Vit B Comp, VitA Alb.
NaValp,Vit B Comp, VitA Alb.
NaValp,Vit B Comp, VitA Alb.
NaValp,Vit B Comp, VitA Alb.
Navalp,VitB12,M/V,Alb

Navalp,Alb,Vit A, Vit B12
Navalp,Alb,Vit A, Vit B12,Mvt
Navalp,Alb,Vit A, Vit B12,Mvt
Phenytoin,Folic Acid
Navalp,Alb,Vit B12,Multivite
Navalp,Alb,Vit B12,Multivite
Navalp,Alb,Vit B12,Multivite
Navalp,Alb,Vit B12,Multivite
Navalp, Alb,Mvt, Vit A

Navalp,Alb,Vit A, Vit B12,Mvt	
Navalp,Mvt,Vit B12	
Navalp CBZ,FA,Vit B Comp	
Navalp,Mvt,Vit B12 Vit A	
Navalp, CBZ,FA,Vit A, B12 Mvt	
Navalp,Vit A, FA, CBZ	
NA valp,VitA, FA,B12,Mvt	Amitted
Navalp, Alb,Vit A,Vit B12	
CBZ, FA,Mvt	
Navalp,Alb,Vit A,B12,Mvt	
Navalp,Vit A, Alb,Vit B12,F/A	
Navalp,Alb, Vit A vit B Comp	
Navalp, CBZ, Vit B 12, Mvt, FA	
Navalp, VitB12, Alb, CBZ,FA	
Navalp,Vit B12 Alb,Vit A	
Navalp,Alb,Vit B12, Vit A,Nutition	
Navalp,Alb,Vita B12	
Navalp,Alb, Vit A, Vit B12	
Navalp,Alb, Vit A, Vit B12	
Navalp,Alb, Vit A, Vit B12	
Navalp, Multivite	
Navalp Multivite	
Navalp,Vit12 inj	
Navalp,Vit A,Alb, inj Vit12	
Navalp, Alb,,Vit A,Vit B Comp, Mvit	
Vit A,Navalp, Vit B12	
Navalp VitA, Alb,BC, Mvt	Admitted
Navalp, Alb,CBZ,Vit A,Vit B Comp, Mvit	Admitted
Navalp, VitB12,M/V	
Navalp, VitB12,M/V	

Navalp, Multivite,Vit B Complex
Navalp, Multivite,Vit B Complex
Navalp, Multivite,Vit B Complex
Navalp, Multivite,Vit B Complex
Navalp, Multivite,Vit B Complex
Navalp, Multivite,Vit B Complex
Navalp, Multivite,Vit B Complex
